# Exosomal miR-373-3p Derived from Docetaxel-Resistant Lung Cancer Cells Targets PDCD4 to Promote Proliferation and Inhibit Apoptosis in Lung Cancer Cells

**DOI:** 10.3390/biomedicines14050986

**Published:** 2026-04-25

**Authors:** Yuan Yuan, Buyi Zhu, Linfei Yang, Yumu Leng, Feifei Chen, Zhenhua Yang, Wei Gu, Kai Zhang

**Affiliations:** 1 Department of Respiration, Nanjing First Hospital, Nanjing Medical University, Nanjing 210006, China; 2 Department of Respiration, Nanjing First Hospital, Nanjing University of Chinese Medicine, Nanjing 210006, China

**Keywords:** lung cancer, exosome, miR-373-3p, PDCD4, docetaxel resistance

## Abstract

**Background:** Lung cancer ranks among the most common and deadly malignant tumors worldwide. Drug resistance is a critical factor hindering the effect of chemotherapy for lung cancer. Exosomes, as intercellular signaling molecule carriers, play an important role in carcinogenesis, metastasis and drug resistance. Our study was aimed at exploring the impact of exosomes derived from docetaxel (DTX)-resistant lung cancer cells on regulating biological behaviors of DTX-sensitive cells, further investigating the molecular mechanisms regarding exosome-mediated intercellular communication. **Methods:** We extracted and identified the exosomes derived from A549, A549/DTX, H1299 and H1299/DTX cells, and then analyzed the expression of exosomal miR-373-3p between DTX-sensitive and DTX-resistant cells. Cell proliferation and apoptosis experiments were verified using a CCK-8 assay, a colony formation assay, a TUNEL assay and flow cytometry. The molecular interaction between miR-373-3p and PDCD4 was evaluated using a dual-luciferase reporter assay. The function of miR-373-3p was further assessed using an in vivo mouse xenograft model. **Results:** We found that the exosomal miR-373-3p level from DTX-resistant A549/DTX or H1299/DTX cells significantly exceeded that from DTX-sensitive A549 or H1299 cells. In addition, both exosomes derived from DTX-resistant lung cancer cells and miR-373-3p mimics could promote the proliferation of DTX-sensitive cells and inhibit their apoptosis. Moreover, we identified PDCD4 as a key target gene of miR-373-3p, which could induce the malignant behaviors of DTX-sensitive cells by reducing PDCD4 expression. **Conclusions:** Our results demonstrated that DTX-resistant lung cancer cells could transfer miR-373-3p to DTX-sensitive cells through exosomes, where miR-373-3p could exert its carcinogenic effect via targeting PDCD4.

## 1. Introduction

As the third most frequently diagnosed cancer, lung cancer represents 10.8% of the global cancer burden. However, its impact on mortality is far greater, causing 28% of all cancer deaths. Looking ahead to 2026, the mortality rate of lung cancer is forecast to be higher than the aggregate of the second- and third-most lethal cancers (colorectal and pancreatic) [1]. As the predominant histological subtype of lung cancer, non-small cell lung cancer (NSCLC) constitutes approximately 85% of all cases [2]. Although various medical treatment modalities, such as targeted therapy, chemotherapy, radiotherapy, and immunotherapy, have been applied, the prognosis of lung cancer patients remains unsatisfactory with the overall survival of less than 15% [3]. At present, cytotoxic chemotherapy remains an effective modality of systemic therapy for most lung cancer patients. However, its clinical efficiency remains limited, owing to the inevitably acquired drug resistance after several cycles of therapy. Therefore, a deeper understanding of the molecular mechanism underlying the pathogenesis of drug resistance in lung cancer is critical for developing effective chemotherapeutic methods.

Exosomes are nanosized (30–100 nm) extracellular vesicles formed from the membrane of multivesicular bodies (MVBs), containing specific cargoes including nucleic acids (DNAs, microRNAs (miRNAs), messenger RNAs (mRNAs), and long non-coding RNAs (lncRNAs)), proteins (transmembrane proteins, cytoskeletal proteins and heat shock proteins), lipids, and so on [4]. Exosomes can transfer the load to the recipient cells and are essential for intercellular communication [5,6,7]. Emerging studies have demonstrated that exosomes play important roles in regulating the initiation, development, metastasis and drug resistance of tumors [8,9,10].

MicroRNA-373-3p (miR-373-3p), located on the chromosomal site of 19q13.42, is a member of the miR-371-3 gene cluster in the miR-520/373 family [11,12]. Emerging evidence has demonstrated that miR-373-3p plays different roles in affecting the invasion, metastasis and proliferation of different cancer cells. Some studies suggested that miR-373-3p was an oncogene in pancreatic cancer [13], prostate cancer [14], colon cancer [15] and colorectal cancer [16], while other investigations demonstrated its tumor-suppressing character [17,18,19]. Therefore, the exact role of miR-373-3p in regulating docetaxel (DTX) resistance of lung cancer remains to be investigated.

As a tumor suppressor, programmed cell death 4 (PDCD4) exerts its anticarcinogenic function by inhibiting protein transcription or regulating nuclear RNA metabolism. Indisputable evidence has shown that PDCD4 contributes significantly to suppressing tumor-promoter-mediated cell transformation [20,21], tumorigenesis [22,23,24,25,26], cell growth [27,28,29,30,31], cell apoptosis [32] and invasion [33,34,35] in various cancers. Moreover, PDCD4 is lost in many tumors like oral squamous cell carcinoma [36], prostate cancer [37], glioma [38] and colorectal cancer [39]. However, its role and molecular mechanism concerning DTX resistance of lung cancer are still unclear.

However, whether docetaxel-resistant lung cancer cells transmit chemoresistance via exosomal transfer of specific miRNAs remains poorly understood. In this study, we hypothesized that DTX-resistant lung cancer cells promote the proliferation and inhibit the apoptosis of DTX-sensitive cells by secreting exosomes enriched with miR-373-3p, which potentially targets the tumor suppressor gene PDCD4. This study aims to investigate the role of the exosomal miR-373-3p/PDCD4 axis in mediating the intercellular transmission of chemoresistance and to evaluate its potential as a therapeutic target for improving lung cancer chemotherapy.

## 2. Materials and Methods

### 2.1. Cell Culture and the Establishment of Docetaxel-Resistant Cells

A549 and H1299 cells were bought from the Cell Bank of the Chinese Academy of Sciences (Shanghai, China). DTX agent was purchased from MedChemExpress (Monmouth Junction, NJ, USA). DTX-resistant cells A549/DTX and H1299/DTX were constructed by continuously exposing A549 and H1299 cells to increasing doses of DTX. In brief, cells of logarithmic phase were exposed to stepwise augmented DTX doses (1 to 1000 μg/L) for 24 h every time. Finally, the surviving cells constituted a DTX-resistant strain, which was preserved with 50 μg/L DTX before use. Cells were cultured in RPMI 1640 medium (Gibco, Grand Island, NY, USA) supplemented with 10% fetal bovine serum (FBS; Gibco) and antibiotics (Gibco), and maintained at 37 °C in a 5% CO_2_ atmosphere.

### 2.2. Exosome Isolation and Purification

Exosomes were collected from the supernatant collected from the culturing medium of each strain by differential centrifugation. To begin, 1 × 10^7^ cells were seeded into 10 cm dishes and allowed to recover for 24 h. After this recovery period, the cells were washed twice using preheated phosphate-buffered saline (PBS). Next, the original medium was removed and replaced with fresh medium containing 10% exosome-free FBS, which had been ultracentrifuged at 100,000 *g* for 8 h. After 3 days of incubation, the culture medium was harvested by sequential centrifugation: first at 2000 *g* for 10 min at 4 °C to remove living or dead cells, and then at 10,000 *g* for 30 min to remove debris. The final supernatant was then pelleted by ultracentrifugation at 100,000 *g* for 70 min at 4 °C. At last, the supernatant was discarded, and the pellets were washed with PBS, followed by ultracentrifugation at 100,000 *g* for 70 min to precipitate the exosomes, which were re-suspended in PBS.

### 2.3. Transmission Electron Microscopy (TEM) and Nanoparticle Tracking Analysis (NTA) for Exosomes

The collected exosomes were fixed by 2.5% glutaraldehyde for 2 h. After washing, 20 μL exosome suspension was loaded on a small carbon-coated copper grid (Electron Microscopy Sciences, Hatfield, PA, USA). After excess suspension was removed, the grid was stained with 3% phosphotungstic acid for 1 min. Finally, a transmission electron microscope (Hitachi, Tokyo, Japan) was used to examine the exosomes. The sizes of nanoparticles were analyzed through NTA technology using NanoSight NS300 (Malvern Panalytical, Malvern, UK).

### 2.4. Cell Transfection and Lentiviral Transduction

miR-373-3p mimics, miR-373-3p inhibitor and negative control (miR-NC) were purchased from GeneChem (Shanghai, China). Short hairpin RNAs (shRNAs) against PDCD4 and an overexpression plasmid for PDCD4 were purchased from GenePharma (Shanghai, China). Cells were seeded at a density of 1 × 10^5^ cells per well in 6-well plates. Cells were transfected upon reaching 70% confluence, using Lipofectamine 2000 (Invitrogen, Waltham, MA, USA) for oligonucleotide transfection for 48 h, or using the Lentiviral Package Kit (Sino Biological, Beijing, China) for shRNA or plasmid lentiviral transduction for 72 h with polybrene (5 ng/μL) to enhance transduction efficiency. For exosome treatment, the cell culturing medium was supplemented with extracted exosomes, followed by cultivation for 72 h.

### 2.5. Quantitative Reverse Transcription-Polymerase Chain Reaction (qRT-PCR)

Total RNA was extracted from cells or exosomes with TRIzol Reagent, and the RNA was reverse transcribed into complementary DNA (cDNA) using the RevertAid First Strand cDNA Synthesis Kit (K1622; Thermo Scientific, Waltham, MA, USA) following the manufacturer’s protocol, while the miScript II Reverse Transcription Kit (QIAGEN, Hilden, Germany) was used for miRNA reverse transcription. qRT-PCR was performed on the CFX96 Touch Real-Time PCR Detection System (Bio-Rad, Hercules, CA, USA) using SYBR Green PCR Master Mix (Applied Biosystems, Foster City, CA, USA). U6 RNA was used as an endogenous control. Data were analyzed according to the 2^−∆∆Ct^ method. The sequences of qRT-PCR primers (GenePharma, Suzhou, China) are listed below. miR-373-3p reverse transcription, 5′-CTCAACTGGTGTCGTGGAGTCGGCAATTCAGTTGAGACACCCCA-3′; miR-373-3p forward, 5′-ACACTCCAGCTGGGGAAGTGCTTCGATTTTG-3′; miR-373-3p reverse, 5′-TGGTGTCGTGGAGTCG-3′. PDCD4 forward, 5′-TGGGCCAGTTTATTGCTAGAG-3′; PDCD4 reverse, 5-ACGCTTTCCACCTTTAGACATAC-3′. U6 forward, 5′-CTCGCTTCGGCAGCACA-3′; U6 reverse, 5′-AACGCTTCACGAATTTGCGT-3′.

### 2.6. Western Blot Analysis

After protein extraction by radioimmunoprecipitation assay (RIPA) lysis buffer (Solarbio, Beijing, China), the BCA Protein Assay Kit (KeyGEN Biotech, Nanjing, China) was used to determine the protein concentration. Before electrophoresis, proteins were mixed with Tris-HCl buffer containing sodium dodecyl sulfate (SDS) and 2-mercaptoethanol and boiled at 95 °C for 10 min. Next, proteins were separated by 10% SDS-polyacrylamide gel electrophoresis (SDS-PAGE), and transferred to polyvinylidene fluoride (PVDF) membranes (p2438; Sigma-Aldrich, St. Louis, MO, USA). To block non-specific binding, the membranes were treated with 5% bovine serum albumin (BSA; Thermo Scientific) for 1 h at ambient temperature. Then membranes were exposed to primary antibodies overnight at 4 °C, followed by washing with Tris-buffered saline containing Tween-20 (TBST) for 5 min × 5 times. Corresponding goat anti-rabbit secondary antibody (ab6721, 1:2000; Abcam, Cambridge, MA, USA) was added, and membranes were incubated for 2 h at room temperature. An enhanced chemiluminescence (ECL) detection system (Amersham Pharmacia, Piscataway, NJ, USA) was applied to analyze the signals. We took Calnexin and GAPDH as the internal reference. The rabbit anti-human primary antibodies (Abcam) were listed below: anti-CD9 (ab92726, 1:2000), anti-CD63 (ab134045, 1:1000), anti-TSG101 (ab125011, 1:1000), anti-calnexin (ab133615, 1:1000), anti-Bax (ab32503, 1:1000), anti-Bcl-2 (ab182858, 1:2000), anti-Bcl-XL (ab178844, 1:20,000), anti-GAPDH (ab181602, 1:10,000), and anti-Calnexin (ab22595, 1:10,000).

### 2.7. Cell Proliferation Assay

Cells were seeded in 96-well plates at 37 °C for 24, 48 or 72 h of culturing. Then, each well was supplemented with 20 μL of the Cell Counting Kit-8 (CCK-8) reagent (Dojindo Laboratories, Kumamoto, Japan). After 2 h of culturing, we measure the optical density using a microplate reader (BioTek, Winooski, VT, USA) at 450 nm (OD450), representing cell viability.

### 2.8. Colony Formation Assay

Cells were seeded in 6-well plates at 100 cells/well and cultured for 2 weeks until colonies could be observed with the naked eye. Colonies were first incubated in 4% paraformaldehyde for 5 min for fixation. They were then stained with a crystal violet solution (0.1% in 20% methanol) for 15 min. The number of colonies with more than 50 cells was counted.

### 2.9. TUNEL Assay

We used terminal deoxynucleotidyl transferase-dUTP nick end labeling (TUNEL) to evaluate cell apoptosis. After paraformaldehyde fixation, cells were incubated with TUNEL reaction buffer in the dark according to the protocol of the TUNEL Assay Kit (Beyotime, Shanghai, China), and nuclei were stained with 4′,6-diamidino-2-phenylindole (DAPI; Invitrogen). The ratio of cells with TUNEL was measured using a microscope (Olympus, Tokyo, Japan).

### 2.10. Flow Cytometry

We used Annexin-V-FITC/PI Apoptosis Detection Kit (Vazyme, Nanjing, China) for flow cytometry to detect cell apoptosis. Cells were collected, resuspended with binding buffer, stained with 5 μL of Annexin V-fluorescein isothiocyanate (FITC) and 5 μL of propidium iodide (PI), and incubated in the dark at room temperature for 30 min. Subsequently, samples were analyzed with a FACScan flow cytometer (BD Biosciences, San Jose, CA, USA).

### 2.11. Dual-Luciferase Reporter Assay

The 3′-untranslated region (UTR) sequence of PDCD4 mRNA was cloned into pmirGLO Dual-Luciferase miRNA Target Expression Vector (Promega, Madison, WI, USA), constituting the pmirGLO/3′-UTR-Wild-type (WT) luciferase reporter. The PDCD4 3′-UTR sequence with mutant (Mut) miR-373-3p binding site, which was established by the QuikChange Site-Directed Mutagenesis Kit (Stratagene, La Jolla, CA, USA), was adopted to construct pmirGLO/3′-UTR-Mut luciferase reporter. Cells were co-transfected with pmirGLO, pmirGLO/3′-UTR-WT or pmirGLO/3′-UTR-Mut, and miR-NC or miR-373-3p mimics. The Dual-Luciferase Reporter Assay System (Promega) was applied to analyze the ratio of firefly/Renilla luciferase activity for each group.

### 2.12. Animal Study

The Institutional Animal Care and Use Committee of Nanjing First Hospital of Nanjing Medical University (Nanjing, China) approved the animal experiments. Female BALB/c nude mice at 4 weeks old (Vital River Laboratory, Beijing, China) were randomly divided into experimental and blank groups. To establish tumor xenografts, the mice in the experimental group were injected with 1 × 10^6^ H1299/DTX cells transfected with a miR-373-3p inhibitor into the right flanks, while those in the blank group were injected with 1 × 10^6^ H1299/DTX cells transfected with miR-NC. Tumor size was measured on days 4, 8, 12, 16, 20, 24 and 28 after inoculation. Tumor volume was estimated using the following formula: volume = (length × width^2^)/2. The mice were anesthetized with diethyl ether. After 4 weeks, we killed mice by cervical dislocation, and tumors were resected and weighed.

### 2.13. Hematoxylin and Eosin (H&E) Staining and Immunohistochemistry (IHC) Analysis

Tumor tissue samples were sectioned by Rotary Microtome (Leica, Frankfurt, Germany), followed by paraformaldehyde fixation and paraffin embedding. Before staining, tissue sections were deparaffinized by xylene for 20 min at 37 °C. The H&E Staining Kit (Sangon Biotech, Shanghai, China) was subsequently applied. For IHC analysis, after heat-mediated antigen retrieval for 20 min, deparaffinized sections were subjected to incubation with rabbit anti-human Ki67 (ab92742, 1:10; Abcam) overnight at 4 °C. Then after 30 min of horseradish peroxidase-labeled secondary antibody treatment, diaminobenzidine tetrahydrochloride (Sigma-Aldrich) was applied for visualization by a microscope (Olympus).

### 2.14. Statistical Analysis

All statistical analyses were carried out using SPSS version 21.0 (SPSS Inc., Chicago, IL, USA). All quantitative values are shown as mean ± standard deviation, calculated from three independent experiments. We used Student’s *t*-test to compare differences between two groups, and used one-way analysis of variance (ANOVA) to compare differences among multiple groups. A difference of *p* < 0.05 was regarded as the threshold of statistical significance.

## 3. Results

### 3.1. Docetaxel-Resistant Lung Cancer Cells Secrete Exosomes with High Levels of miR-373-3p

**Figure 1:**
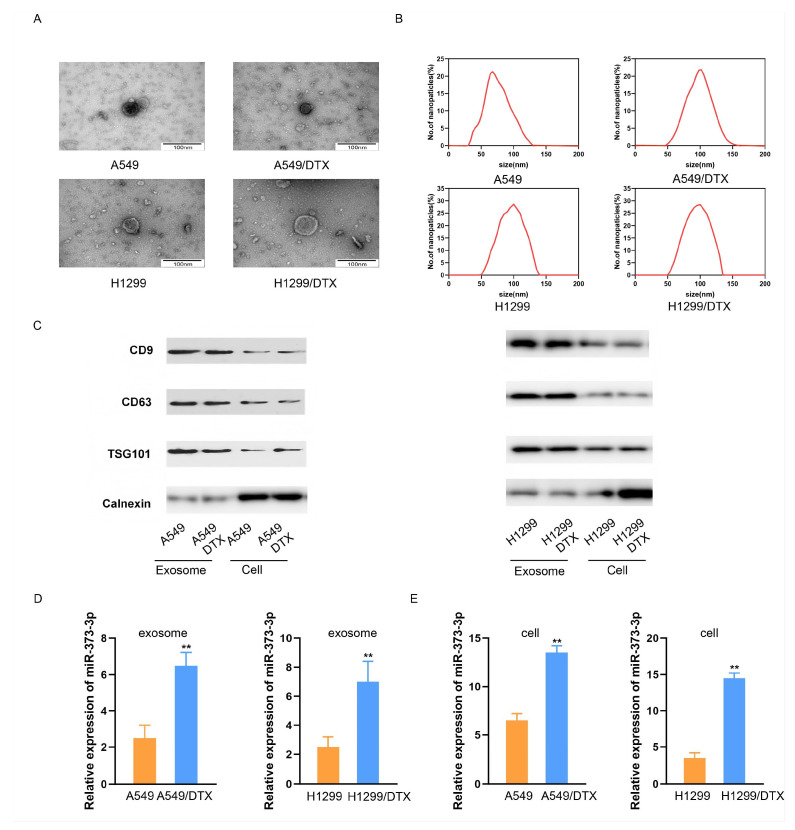
DTX-resistant lung cancer cells secrete exosomes with high levels of miR-373-3p. (**A**) Extracellular vesicles from A549, A549/DTX, H1299 and H1299/DTX cells were observed by TEM. Scale Bars: 100 nm. (**B**) Size distribution of extracellular vesicles from each strain of cells was analyzed by NTA. (**C**) Exosome surface marker proteins CD9, CD63, TSG101 and negative marker calnexin in each group of exosomes and host cells were detected by Western blot analysis. (**D**) The levels of miR-373-3p in exosomes derived from A549 and A549/DTX cells, or in H1299 and H1299/DTX cells, were detected by qRT-PCR. ** *p* < 0.01. (**E**) The levels of miR-373-3p in A549 and A549/DTX cells, or in H1299 and H1299/DTX cells, were detected by qRT-PCR. ** *p* < 0.01.

Extracellular vesicles were isolated from the culturing medium of A549, A549/DTX, H1299 and H1299/DTX cells. TEM showed the morphological characteristics of the circular or elliptical membranous vesicles (Figure 1A). The result of NTA illustrated that vesicle diameters were mostly 50–150 nm (Figure 1B). Moreover, the expression of exosome markers, including CD9, CD63, and TSG101, in the isolated vesicles was examined/analyzed by Western blotting and was higher than in their host cells, while the expression of Calnexin was lower, indicating that exosomes were successfully isolated (Figure 1C). Next, we compared the differential expression of miR-373-3p between exosomes derived from A549/DTX and A549 cells, or from H1299/DTX and H1299 cells. Intriguingly, miR-373-3p was significantly highly expressed in exosomes from both DTX-resistant cells compared with those from parental DTX-sensitive cells (Figure 1D). Additionally, we also compared miR-373-3p levels in the host cells of exosomes. The results of qRT-PCR illustrated the higher expression of miR-373-3p in A549/DTX and H1299/DTX cells than their parental cells (Figure 1E). Therefore, we hypothesized that miR-373-3p was connected with DTX resistance in lung cancer cells.

### 3.2. Exosomes Derived from Docetaxel-Resistant Lung Cancer Cells Affect the Biological Functions of Docetaxel-Sensitive Cells

**Figure 2:**
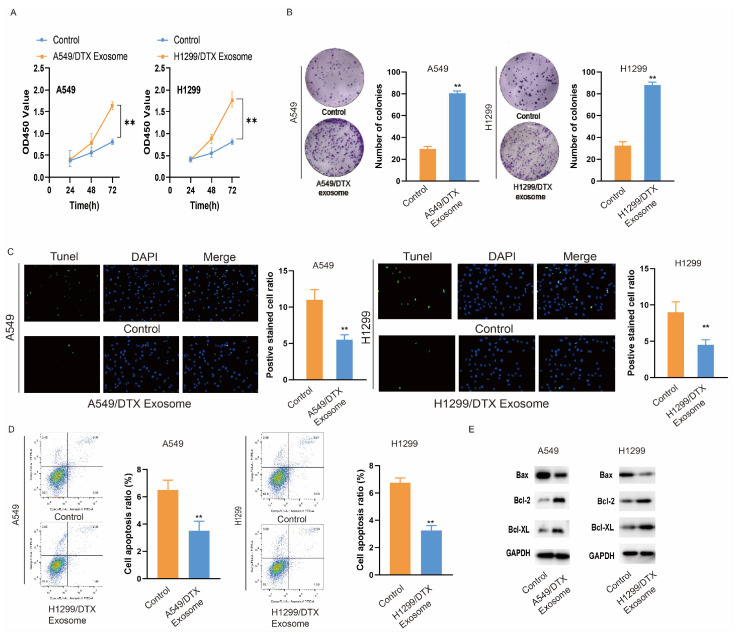
Exosomes derived from DTX-resistant lung cancer cells affect the biological functions of DTX-sensitive cells. (**A**) The proliferation capacity of A549 or H1299 cells with or without exosomes from A549/DTX cells or H1299/DTX cells was detected by CCK-8 assay. (**B**) Colony formation assay was used to examine the number of colonies in each group. (**C**) The apoptotic level of A549 or H1299 cells co-cultured with exosomes from DTX-resistant cells or the control group was assessed using the TUNEL assay. (**D**) We detected cell apoptosis by flow cytometric analysis for each group. (**E**) The expression of Bax, Bcl-2 and Bcl-XL proteins in A549 and H1299 cells was, with or without exosome induction, assessed by Western blot analysis. ** *p* < 0.01.

Subsequently, we explored the influence of exosomes transferring from DTX-resistant lung cancer cells to DTX-sensitive cells. The exosomes secreted by A549/DTX and H1299/DTX cells were isolated and added to the culturing medium of A549 and H1299 cells (10 mg/mL), respectively. After 72 h of exosome induction, the proliferation ability of A549 and H1299 cells increased (Figure 2A). The colony formation assay also verified that exosome stimulation may enhance the growth of lung cancer cells. (Figure 2B). As shown in Figure 2C, after exosome treatment of A549 or H1299 cells, the ratio of apoptotic cells was lower than that in the control group. Flow cytometry also revealed that co-culturing with exosomes from DTX-resistant cells could inhibit the apoptotic level of DTX-sensitive cells (Figure 2D). In addition, the expression of pro-apoptotic protein Bax and anti-apoptotic proteins Bcl-2 and Bcl-XL was also detected by Western blot. After exosome induction, the expression of Bax declined, while the expression of Bcl-2 and Bcl-XL increased (Figure 2E). These results notably demonstrated that the exosomes secreted by A549/DTX and H1299/DTX cells could significantly promote the proliferation abilities of DTX-sensitive cells and inhibit their apoptotic levels, suggesting the involvement of exosomes in the intercellular communication of lung cancer cells.

### 3.3. miR-373-3p Enhances the Proliferation and Suppresses the Apoptosis of Lung Cancer Cells

**Figure 3:**
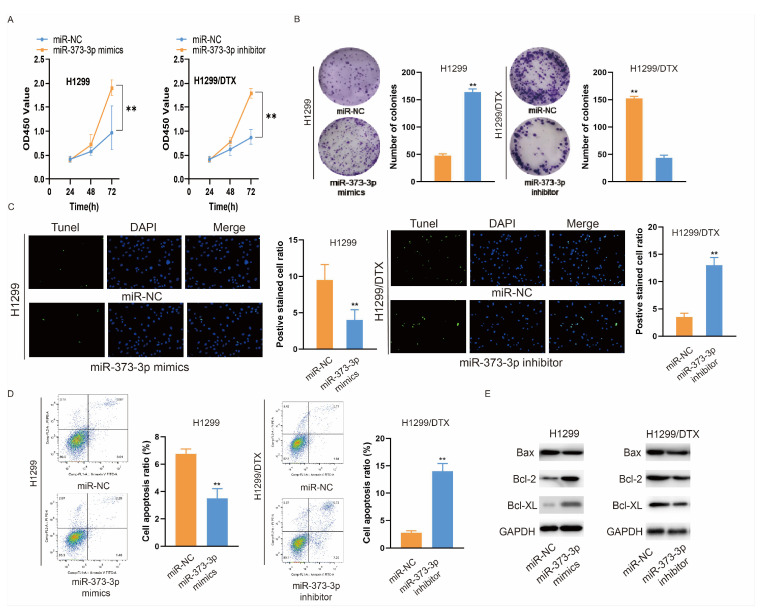
miR-373-3p enhances the proliferation and suppresses the apoptosis of lung cancer cells. (**A**) CCK-8 assay evaluated the proliferation of H1299 cells transfected with miR-373-3p mimics or miR-NC, as well as of H1299/DTX cells transfected with miR-373-3p inhibitor or miR-NC. (**B**) Colony formation assay was performed to examine the number of colonies of H1299 cells and H1299/DTX cells in each group. (**C**,**D**) TUNEL assay (**C**) and flow cytometry (**D**) were used to detect the apoptosis of H1299 cells transfected with miR-373-3p mimics or miR-NC, as well as of H1299/DTX cells transfected with miR-373-3p inhibitor or miR-NC. (**E**) Western blotting was employed to measure the expression of pro-apoptotic protein Bax and of anti-apoptotic proteins Bcl-2 and Bcl-XL in each group. ** *p* < 0.01.

Since miR-373-3p had been identified to be highly expressed in DTX-resistant lung cancer cells and in their exosomes, we explored its role in regulating cell proliferation and apoptosis by transfecting H1299 or H1299/DTX cells with miR-373-3p mimics or miR-373-3p inhibitors, respectively. As illustrated in Figure 3A, the proliferation of H1299 cells transfected with miR-373-3p mimics increased compared with those transfected with miR-NC, while the proliferation of H1299/DTX cells transfected with the miR-373-3p inhibitor decreased compared with the miR-NC group. The colony formation assay showed the same tendency (Figure 3B). Next, we examined whether miR-373-3p influences apoptosis in H1299 cells. Figure 3C,D illustrated that miR-373-3p mimics inhibited the apoptosis of H1299 cells, while the AmiR-373-3p inhibitor promoted the apoptosis of H1299/DTX cells. Additionally, the expression of proteins associated with apoptosis was examined. A Western blot suggested that the expression of Bax was reduced in H1299 cells transfected with miR-373-3p mimics, while the expression of Bcl-2 and Bcl-XL was increased. On the contrary, the expression of Bax was enhanced in H1299/DTX cells transfected with a miR-373-3p inhibitor, while Bcl-2 and Bcl-XL were down-regulated (Figure 3E). These results indicated that the influence of DTX-resistant cell-derived exosomes was owing to the carcinogenic function of miR-373-3p.

### 3.4. miR-373-3p Inhibits the Expression of Its Target Gene PDCD4

**Figure 4:**
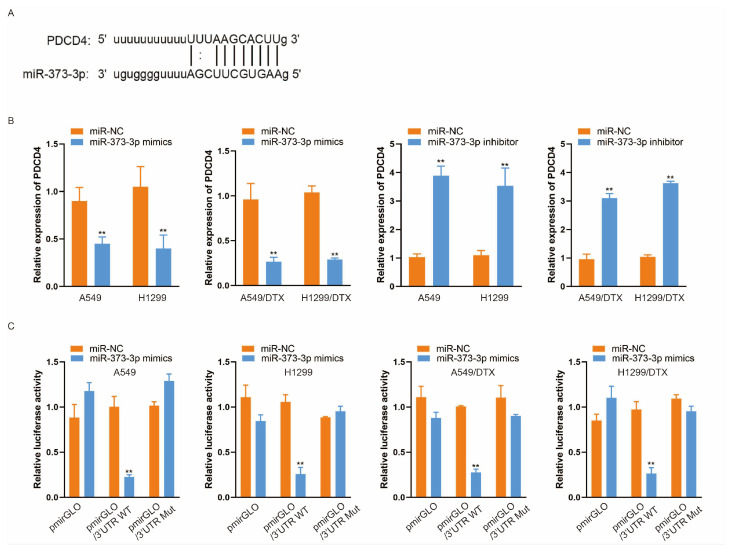
miR-373-3p inhibits the expression of its target gene PDCD4. (**A**) The predicted binding site between PDCD4 and miR-373-3p was shown. (**B**) The expression of PDCD4 in A549, H1299, A549/DTX or H1299/DTX cells transfected with miR-NC, miR-373-3p mimics or miR-373-3p inhibitor was detected by qRT-PCR. (**C**) The luciferase activity of pmirGLO, pmirGLO/3′-UTR-WT and pmirGLO/3′-UTR-Mut in response to miR-NC or miR-373-3p in each strain of cells in a dual-luciferase reporter assay. ** *p* < 0.01.

To discover the key target gene in lung cancer cells by which miR-373-3p exerted its carcinogenic role, we referred to the TargetScan (accessed on 12 March 2020) (http://www.targetscan.org/vert_72), PITA (accessed on 18 March 2020) (http://genie.weizmann.ac.il/pubs/mir07/mir07_data.html) and miRanda (accessed on 25 March 2020) (http://www.microrna.org/microrna/home.do) databases to select the miR-373-3p target genes. Among the candidates, PDCD4 attracted our interest, because it was acknowledged as a common tumor suppressor gene, and it exhibited the highest predictive value in our bioinformatic search. Figure 4A depicts the potential binding site between the 3′-UTR of PDCD4 mRNA and miR-373-3p. Subsequently, we detected the influence of miR-373-3p on PDCD4 expression in A549, H1299, A549/DTX and H1299/DTX cells. qRT-PCR analysis revealed that PDCD4 declined in response to the transfection of miR-373-3p mimics, while transfecting with miR-373-3p inhibitors could increase PDCD4 expression (Figure 4B). Furthermore, as illustrated in Figure 4C, the dual-luciferase reporter assay showed that in A549, H1299, A549/DTX and H1299/DTX cells co-transfected with pmirGLO/3′-UTR-WT and miR-373-3p mimics, the luciferase activity decreased compared with miR-NC groups, while miR-373-3p mimics had no significant impact on the luciferase activity of pmirGLO/3′-UTR-Mut. These data indicated that PDCD4 was a direct target gene of miR-373-3p.

### 3.5. miR-373-3p Targets PDCD4 to Regulate the Proliferation and Apoptosis of Lung Cancer Cells

**Figure 5:**
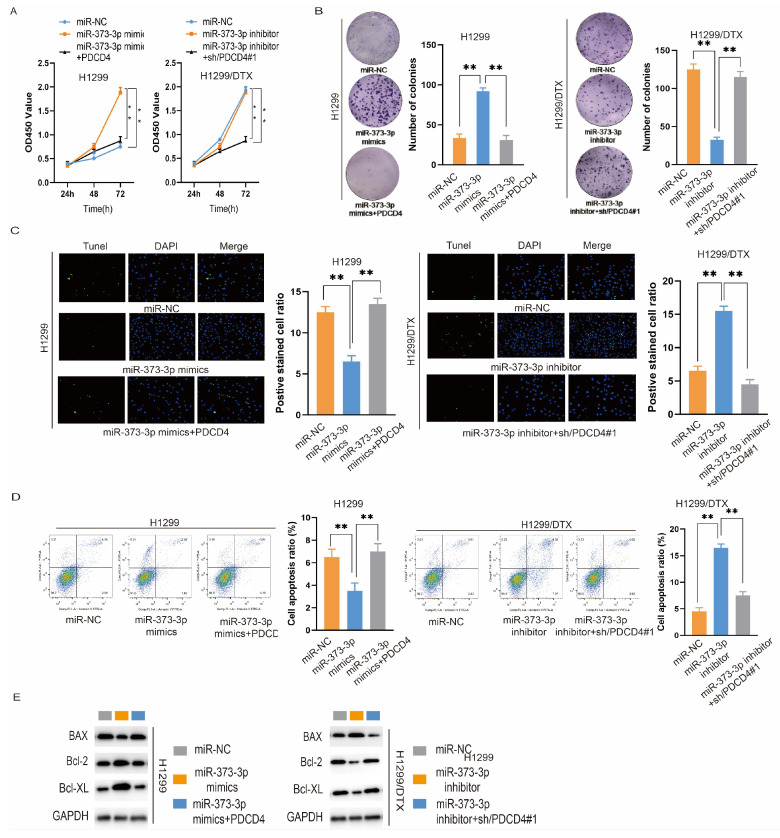
miR-373-3p targets PDCD4, thereby regulating the proliferation and apoptosis of lung cancer cells. (**A**,**B**) CCK-8 assay (**A**) and colony formation assay (**B**) were used to detect cell proliferation in H1299 cells transfected with miR-NC, miR-373-3p mimics or miR-373-3p mimics + PDCD4 overexpression plasmid, or in H1299/DTX cells transfected with miR-NC, miR-373-3p inhibitor or miR-373-3p inhibitor + PDCD4 shRNA. (**C**,**D**) TUNEL assay (**C**) and flow cytometry (**D**) were applied to assess cell apoptosis in each group. (**E**) The expression of Bax, Bcl-2 and Bcl-XL was detected in each group. ** *p* < 0.01.

Next, we examined the role of PDCD4 in regulating miR-373-3p-induced malignant behaviors of lung cancer cells by transfecting a PDCD4 overexpression plasmid or shRNA against PDCD4. Overexpression of PDCD4 could reverse the promotional effect of miR-373-3p mimics on the proliferation of H1299 cells, and PDCD4 knockdown could reverse the inhibitory effect of miR-373-3p inhibitors on the proliferation of H1299/DTX cells (Figure 5A). The colony formation assay showed the same tendency of PDCD4 overexpression or knockdown in regulating the number of colonies of H1299 cells or H1299/DTX cells (Figure 5B). Then we detected the effect of PDCD4 on cell apoptosis. In H1299 cells transfected with miR-373-3p mimics, the reduced apoptotic level was promoted by PDCD4 overexpression. In H1299/DTX cells transfected with miR-373-3p inhibitor, the enhanced apoptotic level was inhibited by PDCD4 knockdown (Figure 5C). In addition, flow cytometry exhibited the same tendency (Figure 5D). Moreover, as shown in Figure 5E, in H1299 cells with miR-373-3p mimics, PDCD4 overexpression restored the down-regulated level of pro-apoptotic protein Bax and the up-regulated levels of anti-apoptotic proteins Bcl-2 and Bcl-XL, while PDCD4 knockdown led to the opposite results in H1299/DTX cells with miR-373-3p inhibitor. In conclusion, we verified that the biological effect of miR-373-3p was mediated through regulating PDCD4.

### 3.6. Down-Regulation of miR-373-3p in Lung Cancer Cells Inhibits Tumor Growth In Vivo

**Figure 6:**
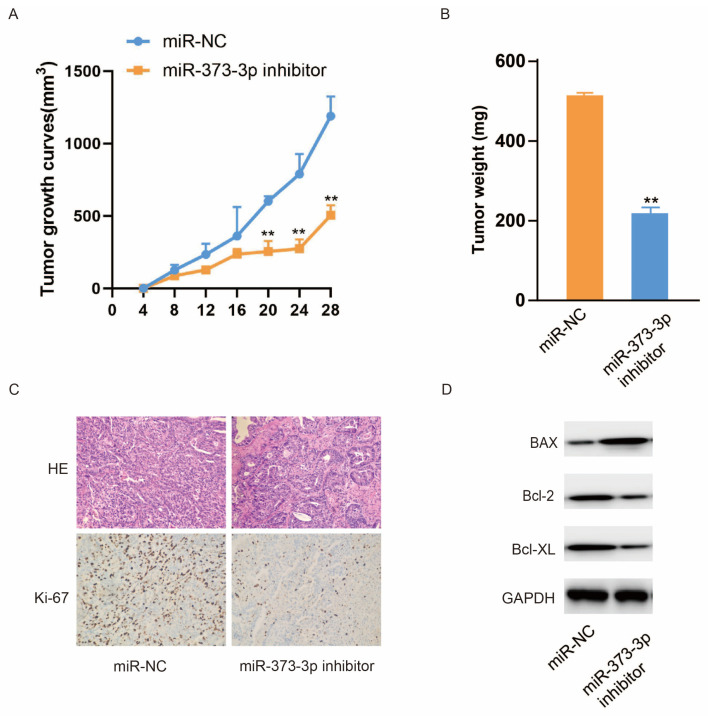
Down-regulation of miR-373-3p in lung cancer cells inhibits tumor growth in vivo. (**A**) The growth curves of tumors of miR-NC group and miR-373-3p inhibitor group in nude mice after transplantation at 4, 8, 12, 16, 20, 24 and 28 days. (**B**) Tumors were resected, and the weight of tumor in each group was measured. (**C**) H&E staining and IHC analysis of Ki67 in the tissue of tumor in each group. (**D**) The expression levels of Bax, Bcl-2 and Bcl-XL in tumor tissues of the miR-NC group and the miR-373-3p inhibitor group were tested by Western blot assay. ** *p* < 0.01.

To verify the role of miR-373-3p in vivo, we constructed a xenograft model in nude mice, which were injected with H1299/DTX cells transfected with miR-NC or miR-373-3p inhibitor, respectively. Figure 6A revealed that tumor growth was slower in the group of the miR-373-3p inhibitor than in the group of miR-NC. In addition, the weight of the tumor was significantly lighter in the group of the miR-373-3p inhibitor (Figure 6B). Subsequently, tumor tissues were subjected to H&E staining and IHC analysis. Figure 6C illustrates that compared with the miR-NC group, the tumor tissue of the miR-373-3p inhibitor group exhibited a lower level of proliferation marker Ki67. Additionally, we detected the levels of apoptosis-related proteins extracted from tumor tissues. Western blot showed that the expression of Bcl-2 or Bcl-XL in the miR-373-3p inhibitor group was lower than that in the miR-NC group, while the expression of pro-apoptotic protein Bax was enhanced (Figure 6D). The results suggested that the tumor growth of lung cancer cells in nude mice could be suppressed by reducing the expression of miR-373-3p.

## 4. Discussion

Chemotherapy is a basic modality for lung cancer. However, the occurrence of drug resistance can limit therapeutic effects, cause cancer recurrence, or even exacerbate tumor progression, thus making the 5-year survival rate and overall survival rate for lung cancer still very low [40,41]. Current studies on the mechanisms of drug resistance in lung cancer include maladjustment of drug efflux genes, enhancement of the repairing function for DNA damage, inhibition of apoptosis, change in tumor microenvironment and so on. Even so, the complicated causes of drug resistance need to be further elucidated. Therefore, a better understanding of molecular mechanisms of drug resistance in lung cancer is beneficial to accurate clinical evaluation, early diagnosis, effective treatment and better prognosis.

Cell-to-cell communication has been traditionally acknowledged to consist of direct cell adhesion [42], the interaction of ligands and receptors [43], as well as the release of soluble factors [44]. Recently, exosomes, as a newfound intercellular communication medium, have been discovered to be able to affect the biological function of adjacent receptor cells. Many studies have shown that exosomes play an important role in regulating the development and drug resistance of tumors. Jing C et al. found that exosomes transported miR-21 to recipient cells to activate the Akt signaling pathway and further lead to gefitinib resistance [45]. . In our study, we isolated the exosomes released from lung cancer cells and verified them by morphological observation, NTA technology and detecting the expression of exosome markers. Moreover, we found that the exosomes from DTX-resistant lung cancer cells could promote the proliferation of sensitive cells and inhibit their apoptosis.

There are two major mechanisms to secrete extracellular miRNAs. One way is through cell apoptosis or necrosis, and the other is to release mature miRNAs contained in exosomes [46,47]. Exosomal miRNAs can reflect intracellular biological changes more accurately and have more significant functions in cell communication than miRNAs without carriers. In 2006, Ratajczak et al. reported that exosomes could transport RNAs among cells and investigated the role of exosomal RNAs in different cells. Many studies have shown that various exosomal miRNAs can be regarded as diagnostic biomarkers and therapeutic targets in lung cancer. As a first-line chemotherapy drug, DTX is widely used in lung cancer. Our study found that the expression of miR-373-3p in exosomes derived from DTX-resistant lung cancer cells was higher than that from sensitive cells, suggesting its connection with lung cancer DTX resistance.

As an oncogene or a tumor suppressor gene in different circumstances, miR-373-3p has been discovered to be abnormally expressed in different tumors, affecting tumor proliferation, metastasis and drug resistance. Aibing WU et al. found that miR-373-3p was significantly up-regulated in lung cancer tissues, and further investigated whether miR-373-3p could up-regulate MMP-9 and MMP-14, thus promoting cell invasion and tumor metastasis [11]. However, the expression of miR-373-3p in prostate cancer tissues was found to be significantly lower than that in normal prostate tissues, and overexpression of miR-373-3p could inhibit the epithelial–mesenchymal transition of prostate cancer cells, thus inhibiting their aggressiveness. In our study, we found that the proliferation abilities of A549 and H1299 cells were enhanced after they were transfected with miR-373-3p mimics, and their apoptotic levels were reduced, resembling the results after the treatment of exosomes derived from DTX-resistant cells, whereas miR-373-3p inhibitor transfection had the opposite effect. These results indicated the carcinogenic effect of miR-373-3p in DTX-sensitive lung cancer cells.

PDCD4, as a tumor suppressor gene, has been reported to be down-regulated in renal cell carcinoma [48], esophageal cancer [49,50], breast cancer [51], skin cancer [51] and many other tumors. The mechanisms by which PDCD4 exerts its important role in tumors mainly comprise three ways: to induce cell apoptosis by inhibiting the expression of Bcl-XL and XIAP or up-regulating pro-apoptotic genes; to inhibit tumorigenesis by inhibiting the translation of Sin1, p53, c-Myb, Bcl-XL and XIAP [52,53,54]; and to inhibit the proliferation and invasion of tumor cells by suppressing the activation of Akt and mTOR [55]. However, the role of PDCD4 in lung cancer is not entirely clear. In our study, PDCD4 was identified as a target gene of miR-373-3p by bioinformatic analysis and dual-luciferase reporter assay. In addition, the overexpression or knockdown of miR-373-3p could reduce or enhance the expression of PDCD4 in lung cancer cells. Moreover, PDCD4 overexpression or knockdown could reverse the function of miR-373-3p mimics or miR-373-3p inhibitors in regulating lung cancer cell proliferation and apoptosis.

It is necessary to acknowledge several limitations of this study. Firstly, generating drug-resistant cell lines in vitro using DTX may not fully capture the complexity of acquired drug resistance in vivo, as this process may introduce heterogeneous resistance mechanisms and genetic drift. Secondly, the changes in growth kinetics observed in these models may affect the interpretation of drug sensitivity analyses. Therefore, although our findings provide mechanistic insights, they need to be validated in more physiologically relevant models in future studies, such as patient-derived xenografts (PDX).

## 5. Conclusions

In conclusion, our study provides evidence that exosomes derived from DTX-resistant lung cancer cells transport miR-373-3p to DTX-sensitive cells, and exosomal miR-373-3p acts as an oncogene to suppress the expression of PDCD4, thus promoting cell proliferation and inhibiting apoptosis in vitro and in vivo. These findings elucidate the mechanism of the miR-373-3p/PDCD4 axis in lung cancer, and suggest exosomal miR-373-3p as a potential target connected with lung cancer drug resistance. Our study may provide a new insight for improving lung cancer chemotherapy.

## Data Availability

The datasets used during the study are available from the corresponding author on reasonable request.
